# A Universal Approach to Enhancing Silicon Hot‐Carrier Photodetectors for CMOS‐Compatible SWIR Imaging

**DOI:** 10.1002/advs.75474

**Published:** 2026-04-30

**Authors:** Eui‐Hyoun Ryu, Hyun Woo Ko, Sunghyun Hwang, Nayeon Kim, Ji‐Hwan Son, Sunghyun Han, Paul Hongsuck Seo, Jisoo Hong, Sang‐Jun Kim, Seon Kyu Yoon, Min‐Chul Park, In‐Ho Lee

**Affiliations:** ^1^ Center for Quantum Technology Korea Institute of Science and Technology (KIST) Seoul Republic of Korea; ^2^ Department of Materials Science and Engineering Korea University Seoul Republic of Korea; ^3^ School of Electrical Engineering Korea University Seoul Republic of Korea; ^4^ Department of Micro/Nano Systems Korea University Seoul Republic of Korea; ^5^ Department of Computer Science and Engineering Korea University Seoul Republic of Korea; ^6^ Hologram Research Center Korea Electronics Technology Institute Seoul Republic of Korea; ^7^ Department of Electrical and Electronic Engineering Yonsei University Seoul Republic of Korea; ^8^ Spatial Optical Information Research Center Korea Photonics Technology Institute (KOPTI) Gwangju Republic of Korea; ^9^ Division of Nanoscience and Technology KIST School at University of Science and Technology Seoul Republic of Korea

**Keywords:** antireflection coating, hot‐carrier photodetector, Schottky diode, SWIR imaging, ultrathin metal film

## Abstract

Silicon hot‐carrier photodetectors offer a CMOS‐compatible pathway for short‐wavelength infrared (SWIR) detection, yet their practical deployment in imaging systems has been constrained by intrinsically low quantum efficiency. Here, we present a universal approach to enhance the quantum efficiency of silicon hot‐carrier photodetectors through the use of a quasi‐generalized antireflection coating (QARC). The QARC design enhances optical absorption by several‐fold in ultrathin metal electrodes that form the metal–silicon Schottky junction, without degrading carrier injection, and is effective regardless of the metal type or electrode thickness. Consequently, a QARC‐integrated hot‐carrier photodetector achieves a responsivity of 7.8 mA/W and an external quantum efficiency of 0.82% at 1310 nm—nearly doubling that of devices without QARC—while maintaining comparable dark current and microsecond‐scale temporal response, as demonstrated using an ultrathin copper electrode. This enhanced quantum efficiency enables the first demonstration of SWIR imaging using a CMOS‐compatible silicon hot‐carrier photodetector, offering higher signal levels and sharper features under low illumination. The silicon hot‐carrier photodetector with QARC offers a promising route toward practical, CMOS‐compatible SWIR imaging sensors.

## Graphical abstract

Silicon hot‐carrier photodetectors offer a CMOS‐compatible pathway for SWIR detection but suffer from intrinsically low quantum efficiency. Here, we introduce a quasi‐generalized antireflection coating (QARC) that universally enhances optical absorption and quantum efficiency, enabling the first CMOS‐compatible SWIR imaging with silicon hot‐carrier photodetectors.

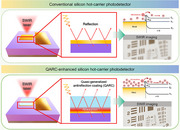

## 1 Introduction

Short‐wavelength infrared (SWIR) imaging has become indispensable in diverse applications, including light‐field imaging [1], night vision [2], machine vision [3], and hyperspectral imaging [4]. Conventional SWIR imaging systems rely primarily on photodetectors based on germanium (Ge) or III–V compound semiconductors such as InGaAs [5], InAs [6], and InSb [7], whose bandgap energies closely match the SWIR photon energy range. Despite their strong performance, these materials require fabrication processes that are expensive, limited in wafer size, and often yield non‐uniform devices due to their poor compatibility with standard complementary metal–oxide–semiconductor (CMOS) manufacturing. These constraints significantly hinder large‐area, high‐yield, and cost‐effective deployment of SWIR imaging systems [8].

As an alternative to conventional SWIR photodetectors, silicon hot‐carrier photodetectors based on metal–silicon Schottky junctions have attracted growing interest [9, 10, 11, 12]. These devices detect sub‐bandgap SWIR photons through the internal photoemission (IPE) process, in which hot‐carriers generated in the metal upon optical absorption are emitted over the metal‐semiconductor Schottky barrier to generate photocurrent [13, 14, 15]. While silicon hot‐carrier photodetectors offer a promising route to SWIR imaging that is fully compatible with silicon platforms, their responsivity is fundamentally limited by weak optical absorption in the metal and the intrinsically low efficiency of IPE [12].

To improve injection efficiency, several strategies have been explored including the use of metals with long hot‐carrier mean free paths and the reduction of metal thickness to below the mean free path, thereby increasing the probability that hot‐carriers reach the interface before relaxation [16, 17]. Another major direction involves enhancing optical absorption in the metal through plasmonic nanostructures [18, 19], which can significantly increase hot‐carrier generation. However, plasmonic nanostructures introduce substantial fabrication complexity and may induce damage or defect formation at the silicon surface, leading to increased scattering and trap‐assisted recombination [20]. Moreover, these nanostructures typically rely on noble metals such as gold (Au) and silver (Ag), which are incompatible with standard CMOS processing.

In this study, we present a CMOS‐compatible SWIR photodetector based on a quasi‐generalized antireflection coating (QARC) structure, where an ultrathin metal layer and a dielectric overlayer jointly enhance optical absorption. The QARC concept extends conventional antireflection coating (ARC) theory to metal–semiconductor systems, in which the refractive index of the dielectric overlayer no longer follows the standard design rules governing conventional ARCs. We first introduce the physical basis of QARC in comparison with conventional ARC theory, followed by a detailed discussion of the resulting design principles. We then experimentally characterize the antireflection performance and absorption enhancement enabled by QARC. The concept is applied to a silicon hot‐carrier photodetector, and its performance is evaluated against a reference device. Finally, we demonstrate SWIR imaging using the fabricated silicon hot‐carrier photodetector, confirming the practical utility of the QARC‐enhanced architecture.

## 2 Results and Discussion

### 2.1 Concept of Quasi‐Generalized Antireflection Coating

**FIGURE 1:**
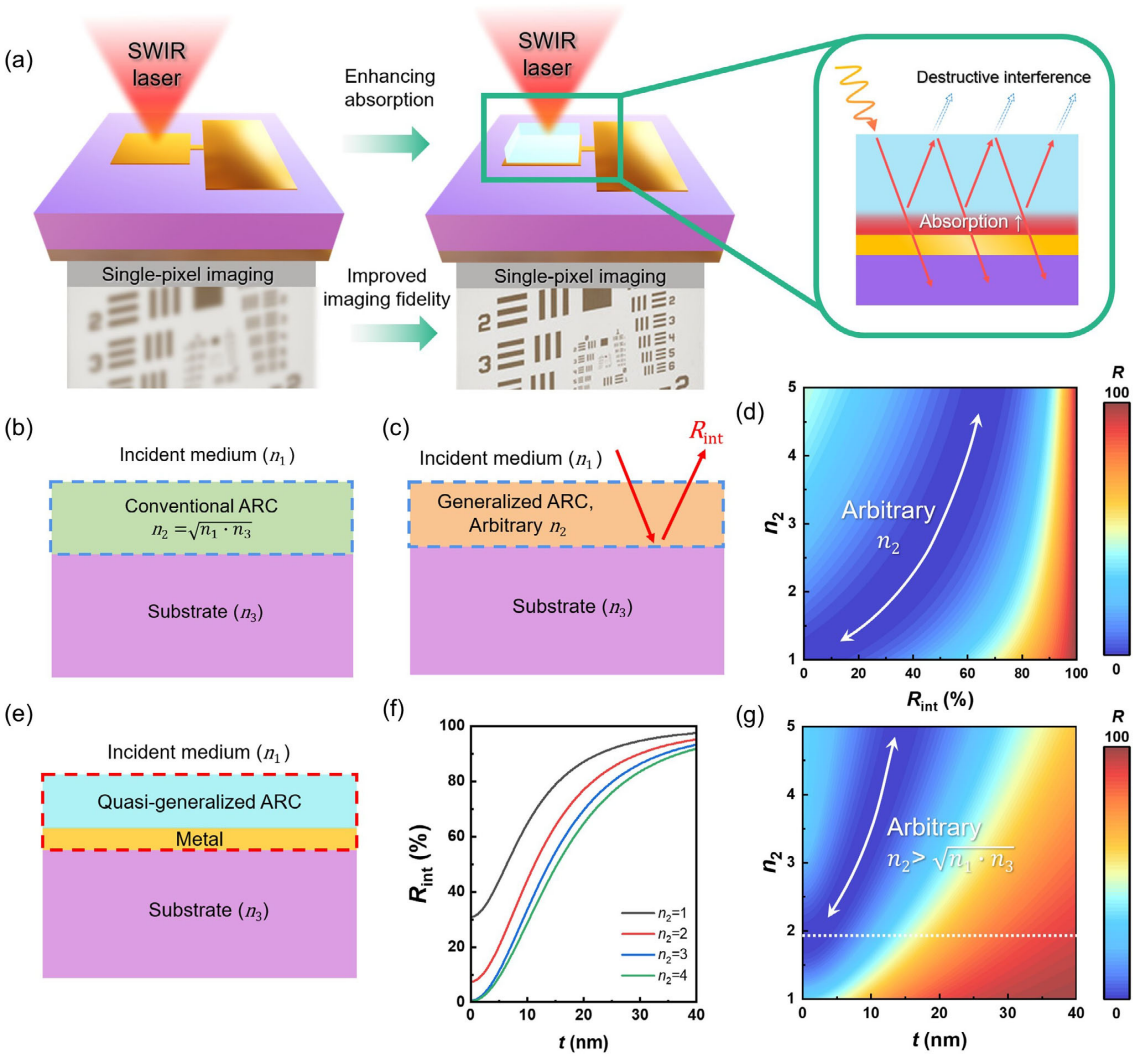
Concept of quasi‐generalized antireflection coating (QARC). (a) Schematic comparison of silicon hot‐carrier photodetector designs and their imaging performance with and without QARC. The inset illustrates the optical interference effects occurring within the QARC structure. (b) Conventional antireflection coating (ARC) model and (c) generalized ARC model, where *n*_1_, *n*_2_ and *n*_3_ denote the refractive indices of the incident medium, the dielectric ARC layer, and the substrate, respectively. (d) Color map of the total reflectance in the generalized ARC structure as a function of the interfacial reflectance *R*_int_ and *n*_2_. (e) Schematic of the proposed QARC configuration. (f) Simulated interfacial reflectance as a function of the metal thickness *t* for several *n*_2_. (g) Color map of the total reflectance of the QARC structure as a function of *t* and *n*_2_, with white dashed lines indicating the region of minimum reflectance.

Instead of employing complex plasmonic nanostructures to enhance hot‐carrier generation, we introduce a QARC to suppress optical reflection and thereby increase optical absorption in the metal. The concept is illustrated in Figure 1a. Because the QARC layer is introduced on top of the pre‐formed metal electrode, it does not directly modify the buried metal–silicon junction and therefore largely preserves the intrinsic junction properties. Therefore, the absorption enhancement introduced by the QARC structure directly leads to an improvement in the external quantum efficiency (EQE) of a silicon hot‐carrier photodetector, as described by the approximate relation EQE ≈ *A*·IQE, where *A* represents the optical absorption and IQE denotes the internal quantum efficiency. Moreover, this approach is universally applicable to silicon hot‐carrier photodetectors with any choice of metal and across a wide range of metal thicknesses. The improved responsivity achieved through QARC leads to higher‐quality images when the detector is used in SWIR imaging.

In order to understand the concept of a QARC, it is instructive to first revisit the conventional ARC model that governs dielectric–dielectric interfaces. Figure 1b illustrates a conventional ARC which is basically a dielectric overlayer with refractive index *n*_2_ inserted between an incident medium (*n*_1_) and a substrate (*n*_3_). In this configuration, the total reflection coefficient is given as [21]:

(1) $$r=\frac{r_{12}+r_{\mathrm{int}}exp\left(-i2k_{2}d\right)}{1+r_{12}r_{\mathrm{int}}exp\left(-i2k_{2}d\right)}$$

where *r*_12_ = (*n*_1_‐*n*_2_)/(*n*_1_+*n*_2_) denotes the Fresnel reflection coefficient at the incident‐medium‐overlayer interface, *k*_2_ is the wavenumber in the overlayer, and *d* is its physical thickness. The term *r*_int_ denotes the Fresnel reflection coefficient at the overlayer‐substrate interface. In the conventional ARC model, *r*_int_ is given as (*n*_2_‐*n*_3_)/(*n*_2_+*n*_3_), which is solely determined by the refractive indices of *n*_2_ and *n*_3_. The total reflectance is minimized when the numerator of Equation (1) approaches zero. This requires that the two components, *r*_12_ and *r*_int_ *exp*(‐*i*2*k*_2_ *d*), have equal magnitudes but opposite phases. The required phase shift is supplied by the propagation term *exp*(‐*i*2*k*_2_ *d*). For lossless media at normal incidence, these magnitude and phase conditions are simultaneously satisfied when

(2) $$n_{2}=\sqrt{n_{1}n_{3}}\;$$

(3) $$d=\frac{\lambda }{4n_{2}}$$

with Equations (2) and (3) being the classical antireflection [22] and quarter‐wavelength condition [23, 24], respectively. This condition ensures destructive interference of reflected light at a specific wavelength λ, yielding ideally zero reflection.

Let us assume that |*r*_int_|^2^ can take arbitrary values, independent of *n*_2_. When this condition is allowed, *n*_2_ is no longer restricted by the conventional ARC relation in Equation (2), as illustrated in Figure 1c, and can take arbitrary values. This extended framework, which we introduce here as the generalized ARC concept, broadens the allowable design space beyond the constraints of the conventional ARC model.

Figure 1d presents a color map of the calculated reflectance based on the generalized ARC concept. The simulation shows that near‐zero reflection can, in principle, be achieved for arbitrary values of *n*_2_ provided that |*r*_int_|^2^ is appropriately selected. In practice, however, real dielectric materials cannot simultaneously provide optical transparency and the required refractive‐index relationship [25].

To realize this concept practically, we introduce QARC composed of an ultrathin metallic film capped by a thin dielectric overlayer as illustrated in Figure 1e. In this configuration, *r*_int_ is determined by the effective reflection coefficient of a metal film sandwiched between the overlayer and the substrate which is given as

(4) $$r_{\mathrm{int}}=\frac{r_{2m}+r_{m3}exp\left(-i2k_{m}t\right)}{1+r_{2m}r_{m3}exp\left(-i2k_{m}t\right)}$$

where *r*_2m_ = (*n*_2_‐*n*_m_)/(*n*_2_+*n*_m_) and *r*_m3_ = (*n*_m_‐*n*_3_)/(*n*_m_+*n*_3_) represent the Fresnel reflection coefficients at the overlayer‐metal and metal‐substrate interface, *n*_m_ is the complex refractive index of the metal, *k*_m_ is the complex wavenumber within the metal, and *t* is the metal thickness. As shown in Figure 1f, *r*_int_ can be now modulated by varying *t* even when *n*_2_ and *n*_3_ are fixed. This tunability relaxes the constraint of Equation (2), allowing *n*_2_ to deviate from the classical ARC value. Because *r*_int_ still depends on *n*_2_ and *n*_3_, this decoupling is incomplete, leading to a reduced tuning range when *n*_2_ is small.

The color map of the total reflectance of a silicon substrate with QARC, calculated using the transfer‐matrix formalism (Figure S1), is shown in Figure 1g. Owing to the tunability provided by the metal thickness, near‐zero reflectance can be achieved over a wide range of *n*_2_ for a given *n*_3_. In the QARC scheme, however, *n*_2_ remains constrained to values larger than $\sqrt{n_{1}n_{3}}$, the refractive index requirement for the conventional ARC. This contrasts with the case of the generalized ARC where *n*_2_ can take arbitrary values. The restraint arises because achieving low interfacial reflectance comparable to the Fresnel reflectance at the air‐overlayer interface with a metal film is impossible when *n*_2_ is smaller than $\sqrt{n_{1}n_{3}}$.

### 2.2 Optical Absorption Enhancement via QARC

**FIGURE 2:**
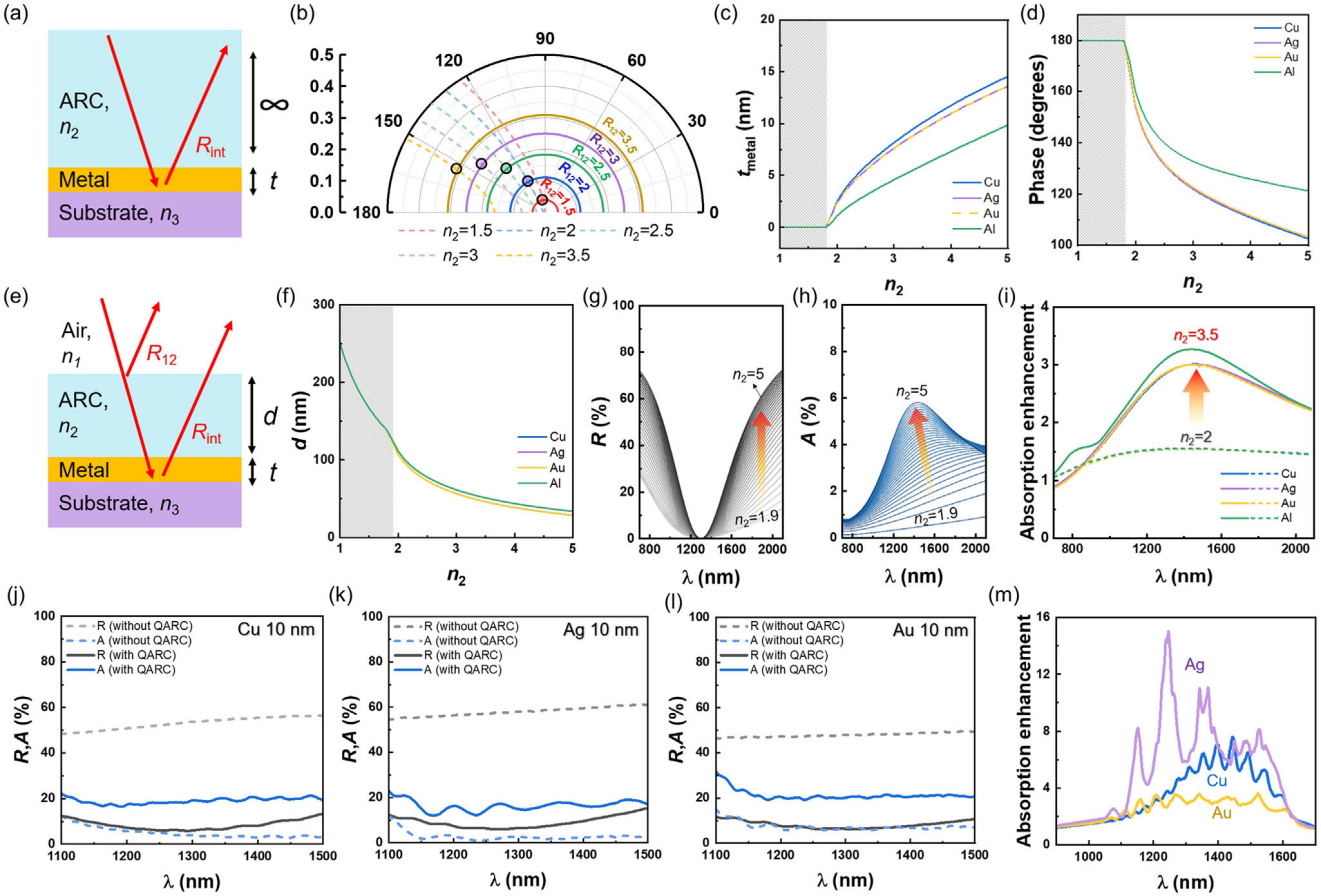
Design principle and absorption enhancement of QARC. (a) Schematic illustration of a metal film sandwiched between two semi‐infinite media with refractive indices of *n*_2_ and *n*_3_. *R*_int_ denotes the effective reflectance of the sandwiched metal film. (b) Calculated *R*_int_ values for metal thicknesses ranging from 0 to 50 nm, plotted in a phasor diagram for several *n*_2_ from 1.5 to 3.5 (dashed lines). The solid lines denote the Fresnel reflectance at the interface of the air and medium with the refractive index of *n*_2_, calculated as [(*n*_2_‐1)/(*n*_2_+1)]^2^. The intersection points between the dashed and solid lines indicate the optimal metal thicknesses. (c) The optimal metal thickness *t*_metal_ as a function of *n*_2_ determined from the intersection points as illustrated in (b) for four representative metals including copper (Cu), silver (Ag), gold (Au), and aluminum (Al). (d) The phase of *r*_int_ as a function of *n*_2_ for the four metals. (e) Schematic illustration of the QARC structure with a finite dielectric overlayer. (f) The optimal overlayer thickness *d* as a function of *n*_2_. (g) Calculated optimal reflection and (h) absorption spectra for a target wavelength of 1300 nm for varying *n*_2_. (i) Absorption enhancement for *n*_2_ = 2 and 3.5. Experimentally measured reflection, transmission, and absorption spectra for QARC‐integrated metal films; (j) Cu, (k) Ag, and (l) Au. (m) Experimental absorption enhancement for each metal (Cu, Ag, and Au).

We now examine the design principles of the QARC stack and its corresponding absorption‐enhancement effects. The analysis begins with a metal film placed between two semi‐infinite media, as illustrated in Figure 2a. Figure 2b presents a phase diagram showing the reflectance of this configuration as a function of *t* for several representative values of the intermediate refractive index *n*_2_. For each *n*_2_, the appropriate *t* is determined by matching the Fresnel reflectance at the air‐overlayer interface with the reflectance of the sandwiched metal film. These solutions correspond to the intersection points in Figure 2b. The resulting relationship between *t* and *n*_2_ for four common metals is summarized in Figure 2c.

Across all materials, the required *t* becomes zero when *n*_2_ is below approximately 1.88, which corresponds to the optimal refractive index for a conventional single‐layer ARC on silicon given by Equation (2). Below this value, the Fresnel reflectance at the air‐overlayer interface is always lower than that at the overlayer‐silicon interface, eliminating the need for a metal layer. This restriction on usable *n*_2_ values represents a key limitation of QARC compared with the generalized ARC model. Using a substrate with a smaller refractive index relaxes this constraint and allows *n*_2_ to span a broader range of values (see Figure S2). Except for aluminum, which has a relatively high optical density, the required metal thicknesses are similar across different metals, with copper (Cu) requiring slightly larger values. For practical refractive indices typically below 4 (the refractive index of Ge), the QARC condition requires *t* below 10 nm. As *n*_2_ increases, the reflection phase change associated with the metal decreases for all metals, with aluminum showing relatively slow variation (Figure 2d).

Based on these reflection phase characteristics, the thickness of the dielectric film can be determined, as shown in Figure 2e. To suppress total reflection, the Fresnel reflection coefficient at the air‐overlayer interface must match the magnitude of the effective reflection coefficient of the metal film but be out of phase, as illustrated in Figure 2d. The overlayer thickness is chosen so that the propagation phase inside the dielectric compensates for the reflection phase difference. The optimal overlayer thicknesses are presented in Figure 2f. Using these values, the optimized reflectance spectra for Cu‐based QARC structures are calculated in Figure 2g. The results show that near‐zero reflectance at 1300 nm is achievable for a wide range of *n*_2_ values, indicating that the practical QARC structure closely approximates the behavior predicted by the generalized ARC model.

This strong suppression of reflectance leads to significantly enhanced optical absorption in the metal layer. Figure 2h shows that the metal absorption peaks near 1400 nm. The deviation from the zero‐reflection wavelength arises from the characteristic increase in the metal's extinction coefficient at longer wavelengths. The enhancement factor relative to a reference device without QARC is shown in Figure 2i. In all cases, optical absorption is enhanced, and both the enhancement magnitude and the prominence of the 1400 nm peak increase with *n*_2_. Our numerical results (Figure S3) support the general applicability of the QARC approach, showing that the absorption enhancement persists across arbitrary complex refractive indices *n*+*iκ* satisfying *n* < *κ* (typical for metals) and metal thicknesses as well as across wavelengths provided that the thicknesses normalized to the wavelength are preserved and *n*_2_ remains constant.

In order to validate the QARC concept, the QARC structures based on Cu, Ag, and Au have been fabricated and characterized. Here amorphous silicon (a‐Si) is chosen as a dielectric overlayer since its high refractive index is beneficial for achieving high absorption enhancement as shown in Figure 2i [26, 27]. To ensure consistent comparison across different metals, the metal‐layer thickness was fixed at 10 nm for all samples. As the extinction coefficient decreases at shorter wavelengths, the optimal value of *n*_2_ decreases accordingly, whereas the optimal thickness *t* increases. The wavelength dependence of these trends is provided in Figures S4 and S5.

Figure 2j–l presents the experimentally measured reflection and absorption spectra for Cu, Ag, and Au films with and without the QARC structure. In all cases, the QARC strongly suppresses reflection while significantly increasing optical absorption in the metal across the SWIR range. This enhancement originates from the spectrally broad QARC resonance, which ensures broadband operation and also imparts tolerance to variations in QARC thickness. The absorption enhancements extracted from the experimental spectra are summarized in Figure 2m. All QARC devices exhibit a clear improvement relative to the devices without QARC, with Ag showing the strongest enhancement, followed by Cu and Au. For Ag and Cu, the measured absorption enhancements significantly exceed the numerical predictions, whereas those for Au are comparable. The higher absorption enhancement than the numerical prediction for Ag and Cu is attributed to additional absorption induced by nanoscale roughness and grain structure in the ultrathin metal films, which increases the actual absorption beyond the idealized model (see film morphology in Figure S6). At comparable thicknesses, Au films tend to exhibit reduced surface roughness and larger grain sizes, allowing their optical response to more closely approach the idealized simulation [28]. The consistent enhancement across three different 10‐nm‐thick metals demonstrates the universality and practical effectiveness of the QARC approach for improving SWIR absorption in ultrathin metal films.

### 2.3 Universal Photoresponse Enhancement

**FIGURE 3:**
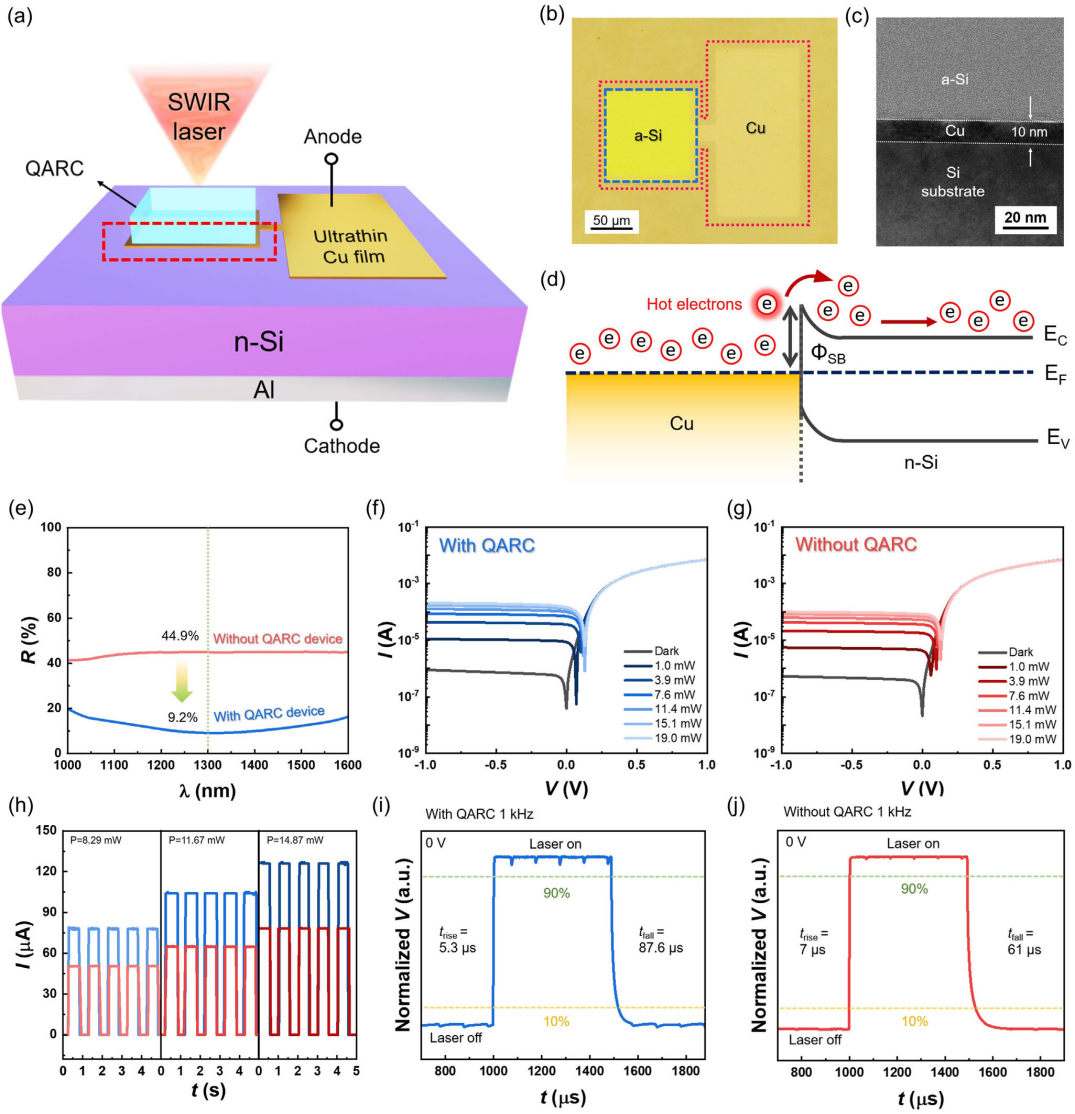
CMOS‐compatible silicon hot‐carrier photodetector with QARC. (a) Schematic illustration of CMOS‐compatible SWIR silicon hot‐carrier photodetector. (b) Optical microscope image of the fabricated device. (c) Cross‐sectional high‐resolution transmission electron microscopy image of the device. (d) Energy‐band diagram illustrating the internal photoemission (IPE) process across the Cu‐Si Schottky interface. (e) Reflectance spectra of devices with and without the QARC. Current–voltage characteristics under SWIR illumination for devices with (f) and without (g) the QARC. (h) Transient photocurrent responses under modulated SWIR illumination. Rise and fall times of devices with (i) and without (j) the QARC.

To experimentally validate the responsivity improvement enabled by the QARC structure, silicon hot‐carrier photodetectors incorporating a QARC structure were fabricated. In the present work, the QARC structure in the fabricated silicon hot‐carrier photodetectors is optimized for operation at 1310 nm. However, the operational wavelength range can be extended to other wavelengths covered by typical silicon hot‐carrier photodetectors, including the visible, near‐infrared, and SWIR regions, by appropriately adjusting the QARC thickness and/or employing dielectric materials that are optically transparent in the corresponding spectral range. Figure 3a schematically illustrates the device architecture. An a‐Si layer is deposited on top of a Cu electrode formed on an n‐type silicon substrate, thereby creating the QARC stack, as illustrated in the fabrication process provided in Figure S7. The choice of a‐Si is motivated by its high refractive index, which, as discussed earlier, enhances QARC‐induced absorption. Given the refractive index of a‐Si, the optimal Cu‐film thickness is determined to be 10 nm based on the relation between *n*_2_ and *t* in Figure 2c. This thickness is also well below the typical mean free path of photocarriers in Cu, thereby increasing the likelihood that carriers generated in the metal reach the metal‐silicon interface and improving injection efficiency [29]. Cu can be deposited at low temperature with high uniformity, exhibits excellent electrical conductivity, and is fully compatible with standard CMOS process flows. These attributes make Cu a practical and scalable choice for implementing NIR hot‐carrier photodetectors [30, 31]. All other constituent materials used in this work are likewise compatible with standard CMOS fabrication processes.

Figure 3b presents an optical microscope image of the fabricated CMOS‐compatible silicon hot‐carrier photodetector. The active device area, where the metal‐silicon Schottky junction and QARC are formed, is approximately 100 µm × 100 µm. Figure 3c shows the cross‐sectional transmission electron microscopy (TEM) image of the Cu film, confirming a uniform thickness of approximately 10 nm. The TEM analysis reveals a continuous and conformal ultrathin metal layer with a well‐defined metal‐silicon interface, confirming that the underlying junction remains physically intact after the deposition of the a‐Si overlayer. The photodetection mechanism is shown in Figure 3d. Upon SWIR illumination, incident photons are absorbed within the ultrathin Cu layer, generating hot electrons through intraband excitation. Electrons possessing sufficient energy to overcome the Schottky barrier (Φ_SB_) are injected across the metal‐silicon interface into the silicon conduction band, producing photocurrent via the IPE process.

Figure 3e presents the experimentally measured reflectance spectra for devices with and without QARC structure. The QARC structure exhibits a marked suppression of reflection—only 9.2% at 1300 nm—compared with 44.9% for the device without QARC. This substantial reduction confirms that the QARC efficiently couples incident photons into the absorbing Cu region. The device characteristics were measured using our confocal optical setup (Figure S8), which efficiently delivers the 1310 nm beam onto the device. Figure 3f,g shows the current–voltage (*I*–*V*) characteristics for various laser powers at 1310 nm. Both devices exhibit clear photocurrent responses, while the device with QARC shows a higher photocurrent.

Since the QARC structure is formed by depositing an a‐Si layer onto the pre‐formed metal‐silicon junction, it does not significantly modify the buried metal‐semiconductor interface. Consistently, the two device types exhibit nearly identical rectification behavior, with a moderate increase in the ideality factor from 1.227 ± 0.010 to 1.358 ± 0.015 for the QARC device. This modest change indicates that the junction property remains largely unaffected, with only a minor contribution from interfacial trap states at the overlayer‐metal junction. The dark current also shows a moderate increase (∼2 ×), which is attributed to enhanced surface leakage associated with the QARC structure. To assess its impact on the photoresponse, this variation can be interpreted as a small effective change in the Schottky barrier height. The extracted barrier heights for the two device types differ by only 0.024 eV, which, based on the Fowler‐type internal photoemission relation at 1310 nm [32], corresponds to an expected IQE variation of only ∼10–15%, assuming that the IPE process is primarily governed by the barrier height. This is significantly smaller than the experimentally observed responsivity enhancement discussed in the following section.

The temporal responses of the devices were examined under a modulated 1310 nm laser at a frequency of 1 Hz. Figure 3h shows the transient photocurrent response, demonstrating stable and repeatable switching for both structures, with the QARC device displaying a larger signal amplitude under zero bias for various laser powers. Figure 3i,j summarize the temporal response that was measured under zero bias condition with incident light modulated at a frequency of 1 kHz using a digital oscilloscope. The QARC device exhibits rise and fall times of 5.3 µs and 87.6 µs, respectively, comparable to 7.0 µs and 61 µs for the reference device. The comparable rise and fall times show that the presence of QARC has no significant impact on the injection process. The observed rise–fall time asymmetry indicates trap states at the metal‐silicon interface, which may originate from surface contamination, native‐oxide residues, or incomplete interfacial cleaning during the metallization step [33]. Overall, these results demonstrate that integrating a QARC with a Schottky junction effectively suppresses reflection, enhances SWIR absorption, and increases photoresponsivity while maintaining low dark current and microsecond‐scale response times. The temporal response of the present devices is primarily limited by the extrinsic RC time constant associated with the large contact pads and the resulting parasitic capacitance, rather than intrinsic hot‐carrier dynamics that occur on femtosecond time scales. Further improvements in response speed can be achieved through device‐level optimization, such as reducing the metal pad area, minimizing parasitic capacitance, and scaling the junction geometry.

**FIGURE 4:**
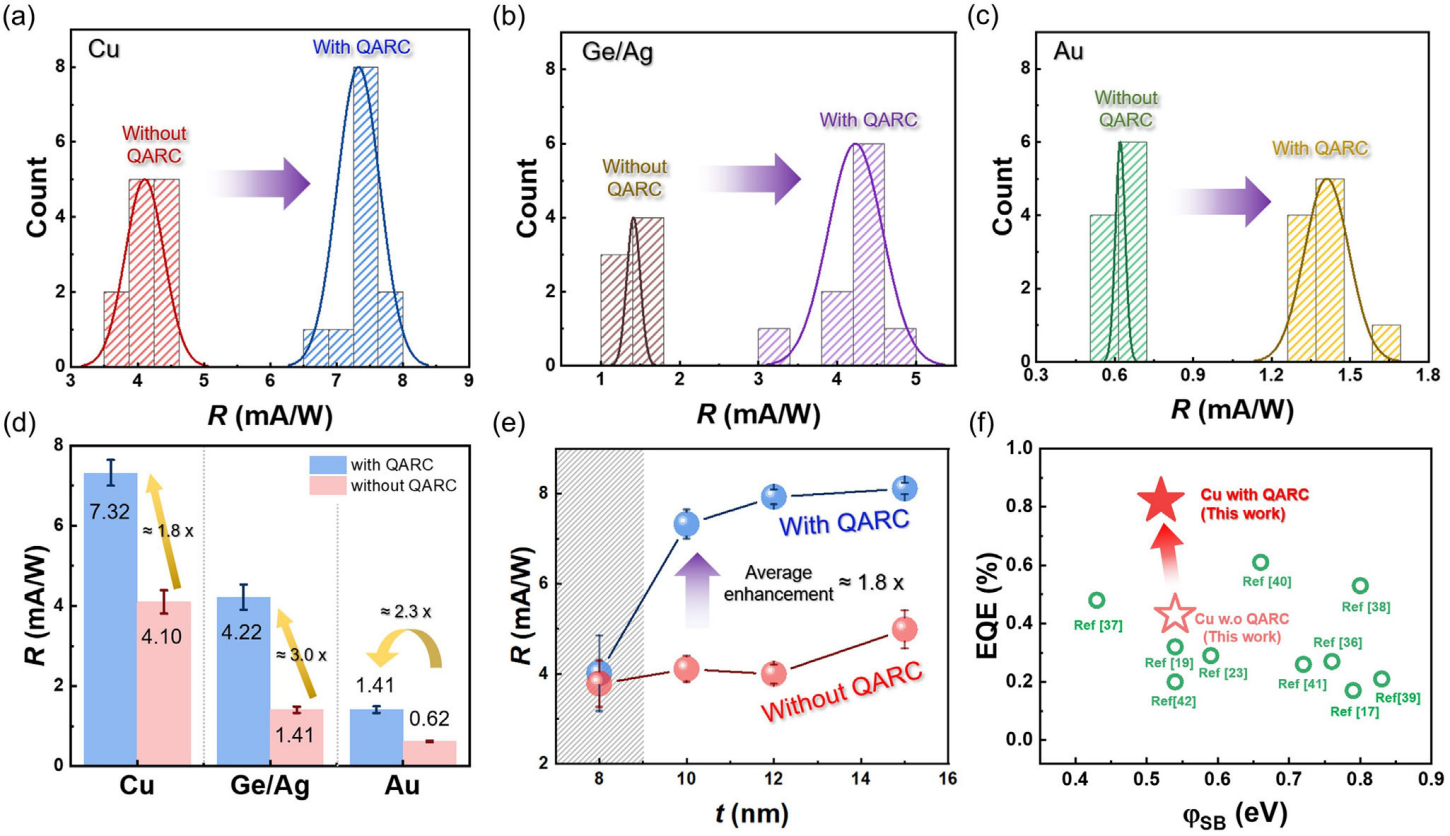
Universal photoresponse enhancement enabled by QARC. (a–c) Statistical distribution of responsivity (*R*) for silicon hot‐carrier photodetectors with different metal electrodes: (a) Cu, (b) Ge/Ag, and (c) Au, comparing devices with and without QARC. (d) Responsivity at 1310 nm for different metal electrodes. (e) Thickness‐dependent responsivity of Cu‐based silicon hot‐carrier photodetectors with and without QARC. (f) Benchmark of external quantum efficiency (EQE) as a function of Schottky barrier height (Φ_SB_) for silicon hot‐carrier photodetectors operating at 1310 nm laser front‐side illumination. For a fair comparison, we include only the cases in which the conducting layer consists solely of metal films.

To further examine the universality of the QARC approach, devices employing different Schottky metals were fabricated and compared. Figure 4a–c presents the statistical distribution of responsivity at zero bias and a wavelength of 1310 nm for silicon hot‐carrier photodetectors employing different metal electrodes, including Cu, Ge/Ag, and Au, with and without the QARC structure. These metals are among the most practical choices for silicon hot‐carrier photodetectors, as they provide relatively high responsivity compared to other commonly used metals. In all cases, the introduction of QARC results in a clear shift of the responsivity distribution toward higher values, indicating consistent enhancement across multiple devices.

Figure 4d summarizes the average responsivities with standard deviations. The Cu‐based device exhibits the highest responsivity among the tested metals, increasing from 4.10 ± 0.31 mA/W to 7.32 ± 0.48 mA/W, while Ag and Au devices also show consistent improvements upon QARC integration. The maximum responsivity obtained for the Cu‐based device reaches 7.8 mA/W (see Figure S9 for the photocurrent power dependence used to extract the responsivity). The superior performance of the Cu‐based device is attributed to its longer hot‐carrier mean free path, which facilitates more efficient carrier transport toward the metal‐silicon interface. It should be noted that a 1‐nm‐thick Ge seed layer is introduced prior to Ag deposition to improve the uniformity and continuity of the ultrathin silver film. The average responsivity enhancement factors, defined as the ratio of responsivity with QARC to that without QARC, are approximately 1.8× for Cu, 3.0× for Ge/Ag, and 2.3× for Au. In general, the responsivity enhancement factors are smaller than the corresponding absorption enhancement factors. This deviation is attributed to increased hot‐carrier thermalization and electric‐field screening at higher hot‐carrier densities [34, 35]. Nonetheless, the consistent enhancement across different metals confirms that the performance improvement enabled by QARC is robust and largely independent of the metal choice.

Figure 4e shows the thickness‐dependent responsivity of Cu‐based silicon hot‐carrier photodetectors with and without QARC. Clear enhancement is observed in the practical ultrathin regime (10–15 nm), where the metal film remains continuous. At 8 nm, the enhancement is reduced due to proximity to the percolation threshold, where film discontinuity weakens the interference effect and increases the series resistance, thereby reducing efficient carrier collection. Therefore, the reduced performance at 8 nm reflects a limitation of film continuity rather than the QARC principle itself. At larger thicknesses, our simulations predict even greater absorption and responsivity enhancement factors. As the metal thickness increases, however, the IQE significantly decreases, limiting the practical thickness range to below 20 nm. Within this practical thickness range, the responsivity enhancement remains consistent, demonstrating the robustness of the QARC approach.

Figure 4f benchmarks the EQE of the present QARC photodetector against various silicon hot‐carrier devices reported in the literature, plotted as a function of Φ_SB_ [17, 19, 23, 36, 37, 38, 39, 40, 41, 42]. A systematic comparison with previously reported silicon hot‐carrier photodetectors, including device structure, Schottky barrier height, responsivity, and EQE, is summarized in Table 1. The Schottky barrier height of our Cu‐based device was extracted using the Cheung‘s method [43], yielding a value of 0.518 eV. Without QARC, the EQE of our device is 0.43%, comparable to previous designs based on ultrathin metal films. With QARC, the EQE increases to 0.82%, outperforming previously reported devices across a wide range of Schottky barrier heights. Notably, the achieved EQE also exceeds those of devices with similar or even smaller Schottky barrier heights, indicating that the enhancement originates from increased optical absorption enabled by QARC rather than barrier lowering. These results demonstrate that the proposed structure effectively overcomes the conventional trade‐off between EQE and Schottky barrier height, offering a simple yet powerful strategy for enhancing SWIR detection efficiency.

**TABLE 1:** Benchmark comparison of silicon hot‐carrier photodetectors operating at 1310 nm.

Metal	Structure	Schottky barrier height (eV)	Responsivity (mA/W)	EQE (%)	Ref.
Cu	Thin film (with QARC)	0.52	7.8	0.82	This work
Cu	Thin film (without QARC)	0.54	4.14	0.43	This work
Au	Au/Si^a^	0.79	1.6	0.17	[17]
Au	Metamaterial Au/Si	0.54	3.0	0.32	[19]
Cu	Cu/Si nanoholes	0.59	2.7	0.29	[23]
Au	Disordered Au/Si nanoneedles	0.76	2.6	0.27	[36]
Al	Al/Si	0.43	4.5	0.48	[37]
Au	Pyramid Si/Au^a^	0.79	5.0	0.53	[38]
Au	Si/TiO_2−x_/Au	0.83	2.0	0.21	[39]
Au	Au/Si nanoholes	0.73	5.8	0.61	[40]
Au	Au/Si nanopillar	0.72	2.5	0.26	[41]
Ti/Au	Nanograting	0.54	1.9	0.20	[42]

^a^ Schottky barrier heights marked with an asterisk were estimated from reported literature values of metal–silicon Schottky junctions, as they were not explicitly provided in the original references.

### 2.4 SWIR Imaging Demonstration

**FIGURE 5:**
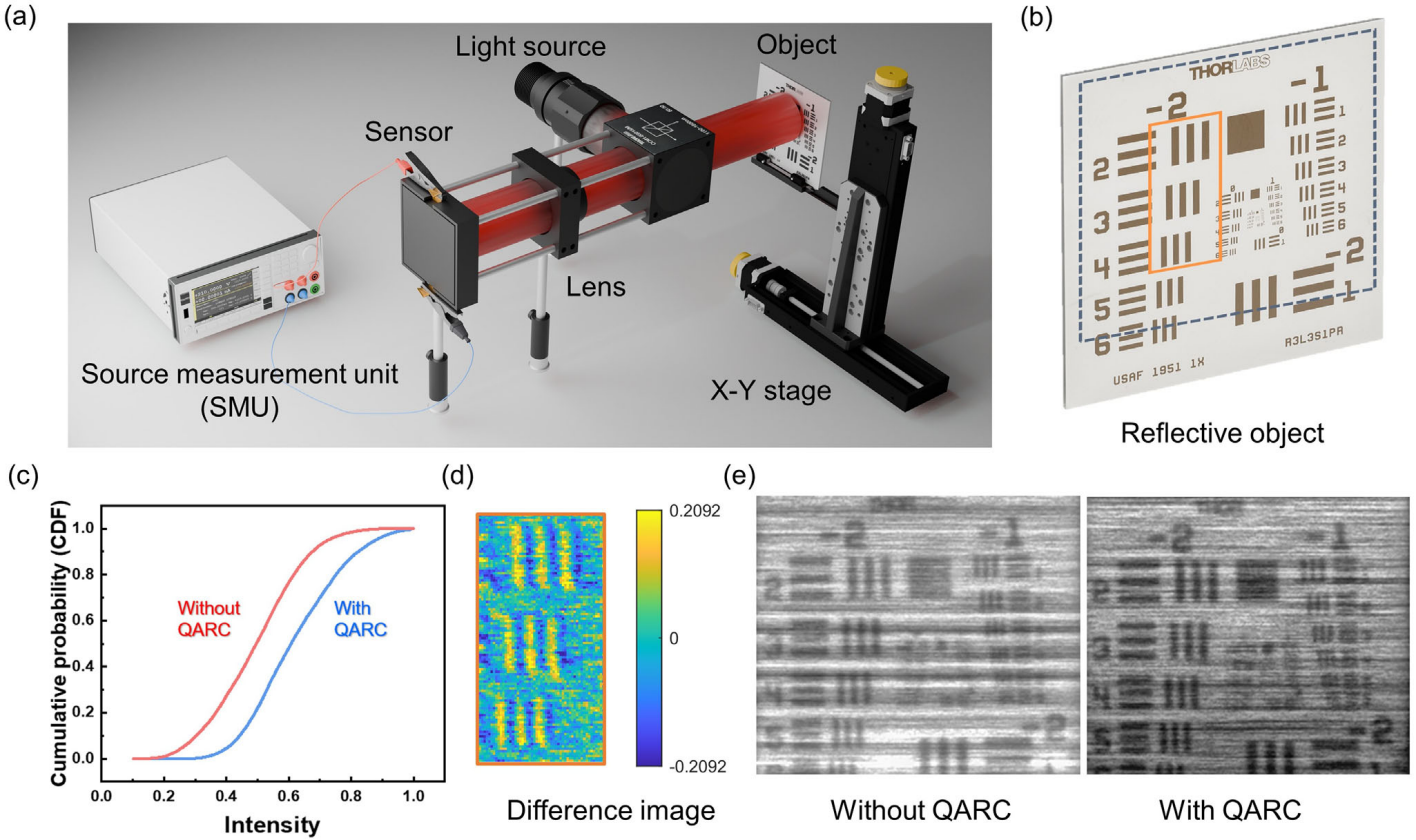
SWIR reflection‐mode imaging and comparison of devices with and without QARC. (a) Reflection‐mode imaging setup using a 1300 nm LED, 50:50 non‐polarizing beam splitter (NPBS), imaging lens, and X‐Y translation stage. (b) USAF‐1951 reflective target; the dashed outline indicates the imaged field of view, and the orange box marks the region used for analysis in (d). (c) Intensity cumulative distribution functions of images from devices with and without QARC. (d) Pixel‐wise difference image (with QARC − without QARC) within the orange region in (b), where positive values correspond to a stronger response from the QARC device. (e) Corresponding processed reflection images from the two devices, displayed using the same global intensity window and post‐processing pipeline.

To further evaluate the imaging performance of the proposed CMOS‐compatible SWIR silicon hot‐carrier photodetector, we performed reflection‐mode imaging at 1300 nm under low‐illumination conditions, as shown in Figure 5a. Light from a 1300 nm LED was directed to a 50:50 non‐polarizing beam splitter (NPBS), transmitted toward a USAF‐1951 reflective target (Figure 5b), and the reflected beam returned through the NPBS to be focused by an f = 35 mm lens onto the device under test (with and without QARC). Device currents were recorded using a source‐measurement unit (SMU) at 0 V external bias. Both devices were measured under identical optical, exposure, and scanning conditions. Images were acquired by raster scanning the target using a bidirectional (snake) line order to reduce stage flyback time while preserving a standard raster pattern [44, 45]. The difference between the conventional and snake raster schemes is illustrated in Figure S10.

For a fair comparison of device response, all images were processed with a single fixed post‐processing pipeline. The minimum and maximum pixel values were first evaluated over the combined dataset (QARC and non‐QARC), and both images were linearly mapped to a shared [0,1] intensity range, ensuring that any apparent brightness differences primarily reflect device responsivity rather than trivial gain or offset. A light row‐wise mean subtraction was then applied to attenuate slowly varying horizontal banding, followed by contrast‐limited adaptive histogram equalization (CLAHE) with identical parameters for both devices to enhance the visibility of fine features (Figure 5e) [46]. The stepwise effect of this post‐processing pipeline on images from both devices is summarized in Figure S11. These latter steps primarily improve readability and do not involve any device‐specific tuning, so the relative contrast and structural differences observed after processing can be attributed to the intrinsic photoresponse of each device.

Under this common normalization and visualization scheme, the QARC device yields visibly sharper bar patterns and stronger micro‐contrast than the non‐QARC reference, particularly in low‐signal and fine‐feature regions. The intensity cumulative distribution function (CDF) for the QARC image is shifted toward higher values compared with the reference image obtained from the same scene and mapped with the same global scaling (Figure 5c), indicating an overall increase in effective signal level—reflected in higher mean and upper quantiles—at equal exposure. The pixel‐wise difference image (QARC—reference), computed after image alignment and row‐wise background subtraction, shows predominantly positive contrast localized along the USAF features with only small residuals in nominally uniform areas (Figure 5d). This pattern demonstrates that the QARC stack enhances structurally meaningful signal across the scene rather than simply elevating the background or amplifying noise.

While SWIR imaging with silicon hot‐carrier photodetectors has been explored only in a few prior studies, those demonstrations relied on non‐CMOS‐compatible metal stacks and transmission‐mode configurations [47, 48]. In contrast, the present work achieves the first SWIR imaging using a fully CMOS‐compatible silicon hot‐carrier photodetector and, moreover, the first reflection‐mode implementation of this imaging modality. Taken together, these reflection‐mode SWIR measurements confirm that integrating the QARC stack into the silicon hot‐carrier photodetector directly translates into improved image quality at equal exposure under photon‐limited conditions. These reflection‐mode SWIR imaging results demonstrate that the QARC stack provides a practical, CMOS‐compatible route to enhanced sensitivity and image quality under photon‐limited conditions, underscoring its potential for real‐world SWIR imaging and sensing applications.

## 3 Conclusion

In summary, we have demonstrated a CMOS‐compatible strategy for enhancing the quantum efficiency of silicon hot‐carrier photodetectors through the implementation of the QARC structure. By integrating an ultrathin metal layer with a dielectric overlayer, the QARC architecture effectively suppresses surface reflection and substantially increases optical absorption in the metal while preserving efficient hot‐carrier injection. Systematic design analysis shows that QARC closely approximates the behavior of a generalized ARC, enabling strong absorption enhancement across a broad range of refractive indices and metal thicknesses. Experimental validation using Cu‐, Au‐, and Ge/Ag‐based devices confirms that QARC consistently improves responsivity, achieving an EQE of 0.82% at 1310 nm for the Cu‐based device while maintaining comparable dark current and microsecond‐scale temporal response. The resulting performance enhancement directly translates into improved SWIR imaging quality, yielding higher effective signal levels, clearer structural contrast, and reduced background noise under low‐illumination conditions. This single imaging demonstration simultaneously represents the first SWIR imaging achieved with a fully CMOS‐compatible silicon hot‐carrier photodetector and the first reflection‐mode implementation of this modality. Collectively, these results establish QARC as a universal and fabrication‐friendly approach for overcoming the inherent absorption constraints of silicon hot‐carrier photodetectors, offering a practical pathway toward high‐performance, large‐area, and CMOS‐compatible SWIR imaging technologies.

## 4 Experimental Section

### 4.1 Device Fabrication

For the fabrication of the QARC structure, a silicon substrate was used. A 10‐nm‐thick Cu film was deposited on the substrate using an electron‐beam evaporator (KVE‐T8897, Korea Vacuum Tech), followed by the deposition of an a‐Si layer as a high‐index overlayer. The thickness of the a‐Si layer was optimized to satisfy the interference condition at the target SWIR wavelength. On the opposite side of the substrate, a 166‐nm‐thick silicon nitride layer was deposited by plasma‐enhanced chemical vapor deposition (PECVD, Plasmalab800Plus, Oxford) to reduce reflection from the back surface.

For the CMOS‐compatible SWIR silicon hot‐carrier photodetector, an n‐type silicon substrate was used. The Schottky junction areas were defined by photolithography, followed by deposition of a 10‐nm Cu layer using the same electron‐beam evaporator. The top Cu film acted as the Schottky contact (anode), while a 100‐nm‐thick aluminum layer was deposited using a thermal evaporator (KVT‐D438, Korea Vacuum Tech) on the back side of the substrate to form the ohmic cathode. An a‐Si overlayer was subsequently deposited on the defined optically active region to serve as the high‐index interference layer.

### 4.2 Device Characterization

The thickness of the deposited metal film was characterized using atomic force microscopy (AFM, XE‐100, Park Systems). Reflectance and transmittance were measured using a spectrophotometer (NIR Quest, Ocean Insight). The current‐voltage characteristics of the silicon hot‐carrier photodetector were measured using a sourcemeter (2636A, Keithley). For the photocurrent measurement, a 1310 nm laser (FPL1053S, Thorlabs) was used to illuminate the device. An oscilloscope (DSOX 1102A, KEYSIGHT) was used to measure the rise and fall times of the silicon hot‐carrier photodetector. A function generator (AFG 31000, Tektronix) was used to apply the required voltage. Reflection‐mode SWIR imaging was performed using a 1300 nm LED (M1300L4, Thorlabs), a 50:50 non‐polarizing beam splitter (NPBS), a USAF‐1951 reflective target (R3L1S1P, Thorlabs), and an f = 35 mm imaging lens, while the photocurrent was measured using a source‐measurement unit (2636A, Keithley).

### 4.3 Numerical Simulation

Throughout the study, the full analytical model we developed has been used to optimize the QARC structure and calculate its transmission spectrum (see Section S1 for details).

## Conflicts of Interest

The authors declare no conflicts of interest.

## Supporting information




**Supporting File**: advs75474‐sup‐0001‐SuppMat.docx.

## Data Availability

Data underlying the results presented in this paper are not publicly available at this time but may be obtained from the authors upon reasonable request.
