# Supplementary material for: A Universal Approach to Enhancing Silicon Hot‐Carrier Photodetectors for CMOS‐Compatible SWIR Imaging
Source: Adv Sci (Weinh). 2026 Apr 30;13(38):e75474. doi: 10.1002/advs.75474 (PMC13335425; doi:10.1002/advs.75474)
Supplement: Supplementary file 1 — Supporting File: advs75474‐sup‐0001‐SuppMat.docx. [file ADVS-13-e75474-s001.docx]

**Supporting Information**

**A universal approach to enhancing silicon hot-carrier photodetectors for CMOS-compatible SWIR imaging**

Eui-Hyoun Ryu, Hyun Woo Ko, Sunghyun Hwang, Nayeon Kim, Ji-Hwan Son, Sunghyun Han, Paul Hongsuck Seo, Jisoo Hong, Sang-Jun Kim, Seon Kyu Yoon, Min-Chul Park* and In-Ho Lee*

E.-H. Ryu, H.W. Ko, S. Hwang, N. Kim, J.-H. Son, S. Han, S.-J Kim, S.K. Yoon, M.-C. Park, I.-H. Lee

Center for Quantum Technology, Korea Institute of Science and Technology (KIST), Seoul, 02792, Republic of Korea

E-mail: inholee87@kist.re.kr, minchul@kist.re.kr

E.-H. Ryu

Department of Materials Science and Engineering, Korea University, Seoul, 02841, Republic of Korea

S. Hwang, N. Kim

School of Electrical Engineering, Korea University, Seoul, 02841, Republic of Korea

J.-H. Son

Department of Micro/Nano Systems, Korea University, Seoul, 02841, Republic of Korea

H.W. Ko, S. Han, P.H. Seo

Department of Computer Science and Engineering, Korea University, Seoul, 02841, Republic of Korea

J. Hong

Hologram Research Center, Korea Electronics Technology Institute, Seoul, 03924, Republic of Korea

S.-J. Kim

Department of Electrical and Electronic Engineering, Yonsei University, Seoul 03722, Republic of Korea

S.K. Yoon

Spatial Optical Information Research Center, Korea Photonics Technology Institute (KOPTI), Gwangju 61007, Republic of Korea

I.-H. Lee

Division of Advanced Materials Science and Engineering, KIST School at University of Science and Technology, Seoul 02792, Republic of Korea

E.-H. Ryu, H.W. Ko and S. Hwang contributed equally to this study.

1. **Full analytical model for quasi-generalized antireflection coating**

**Figure S1** illustrates the geometry of the multilayer overlayer structure used in the analytical calculations. Assuming harmonic time dependence *e^iωt^*, the incident and reflected fields in medium with refractive index *n*_1_ are written as

$$E_{\mathrm{inc}}\left( x \right)=\exp\left( -ik_{0}n_{1}x \right),$$

$E_{\mathrm{ref}}\left( x \right)=r \exp\left( ik_{0}n_{1}x \right),$ (S1)

where *r* is the reflection coefficient and *k*_0_=2π/λ denotes the free-space wavevector. The transmitted field in the substrate *n*_4_ is written as

$E_{\mathrm{tr}}\left( x \right)=t\exp\left( -ik_{0}n_{4}x \right),$ (S2)

where *t* is the transmission coefficient.

In the transfer-matrix formalism, the forward and backward fields in the incident medium and the substrate can be written as column vectors $\left[ \begin{matrix} 1 \\ r \end{matrix} \right]$ and $\left[ \begin{matrix} t \\ 0 \end{matrix} \right]$. The transfer matrices for the individual layers *l* = 1, 2, 3, are given by

$M_{l}=\frac{1}{2}\left[ \begin{matrix} (1+\frac{\gamma_{l+1}}{\gamma_{l}})exp(-ik_{0}n_{l}d_{l}) & (1-\frac{\gamma_{l+1}}{\gamma_{l}})exp(ik_{0}n_{l}d_{l}) \\ (1-\frac{\gamma_{l+1}}{\gamma_{l}})exp(-ik_{0}n_{l}d_{l}) & (1+\frac{\gamma_{l+1}}{\gamma_{l}})exp(ik_{0}n_{l}d_{l}) \end{matrix} \right]$ *l* = 1, 2, 3. (S3)

where γ*_l_* = 1/*n_l_* and the thicknesses are *d*_1_ = 0, *d*_2_ = *d*, *d*_3_ = *t*, *d*_4_ = 0. The overall system matrix is **M** = **M_3_M_2_M_1_**. If

$\mathbf{M}^{-1}=\left( \begin{matrix} a & b \\ c & d \end{matrix} \right)$, (S4)

the boundary condition gives

$t=\frac{1}{a}$, $r=ct$. (S5)

The measurable transmittance and reflectance are given by

*T*=$\frac{n_{4}}{n_{1}}\left| t \right|^{2}$, *R*=$\left| r \right|^{2}$. (S6)

The transmission spectra calculated using this analytical model are identical to those obtained from the full-wave simulations based on the rigorous coupled wave analysis.

To provide a closed-form analytical description of the reflectance of the multilayer structure in Figure S1, we decompose the total reflection into contributions from the air-overlayer interface, the metal film, and the propagation phase inside the dielectric overlayer. The reflection coefficient at the air-overlayer interface is given by *r*_12_ = (*n*_1_–*n*_2_)/(*n*_1_+*n*_2_). The metal layer of refractive index *n*_3_ ​ and thickness *t*, which is sandwiched between the overlayer (*n*_2_) ​ and the substrate (*n*_4_) ​, produces an interfacial reflection coefficient *r*_int_ = [*r*_23_+*r*_34_exp(-*i*2*k*_3_*t*)]/[1+*r*_23_*r*_34_exp(-*i*2*k*_3_*t*)], where *r*_23_ = (*n*_2_–*n*_3_)/(*n*_2_+*n*_3_), *r*_34_ = (*n*_3_​–*n*_4_​)/(*n*_3_​+*n*_4_​), and *k*_3_=(2π/λ)*n*_3_​. Propagation through the dielectric overlayer introduces an additional phase factor determined by *k*_2_ = (2π/λ)*n*_2_ and its thickness *d*. Combining these contributions yields the total reflection coefficient of the complete air-dielectric-metal-substrate system, which is expressed as

$r= \frac{r_{12}+r_{\mathrm{int}}exp\left( -i2k_{2}d \right)}{1+r_{12}r_{\mathrm{int}}exp\left( -i2k_{2}d \right)}$. (S7)

This closed-form expression is used throughout the Supporting Information to compute the reflectance spectra and exactly matches the results obtained from the transfer-matrix model and rigorous coupled-wave simulations.


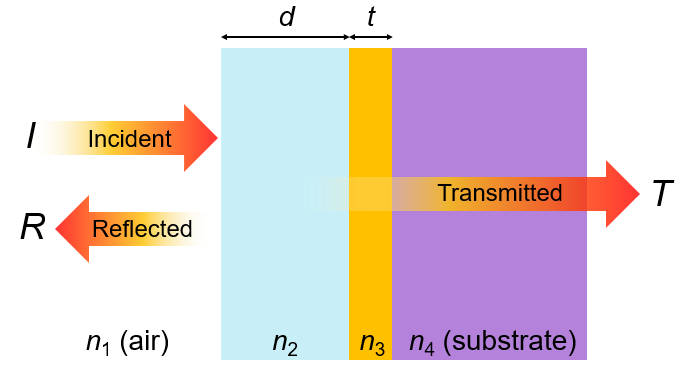


**Figure S1**. Schematic illustration of high-index overlay.


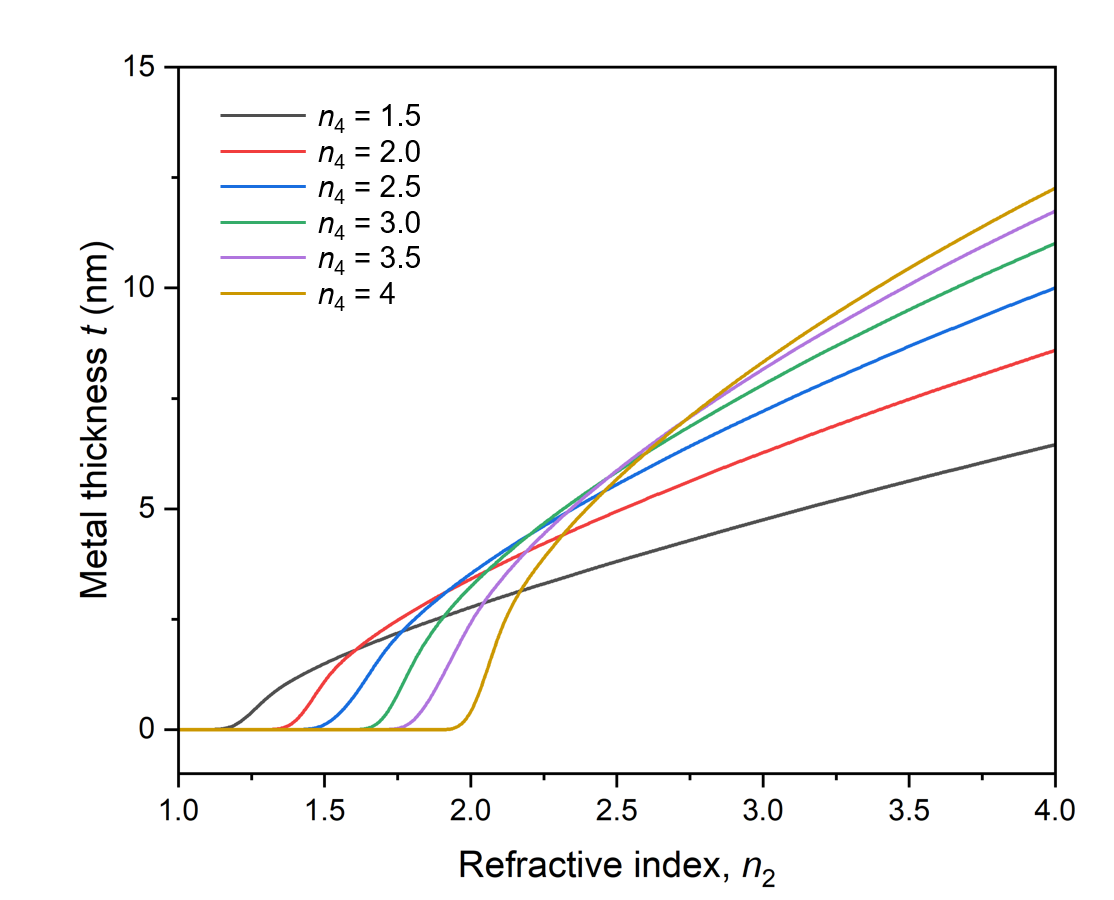


**Figure S2**. Optimal metal thickness *t* as a function of *n*_2_ for copper on substrates with different refractive indices.


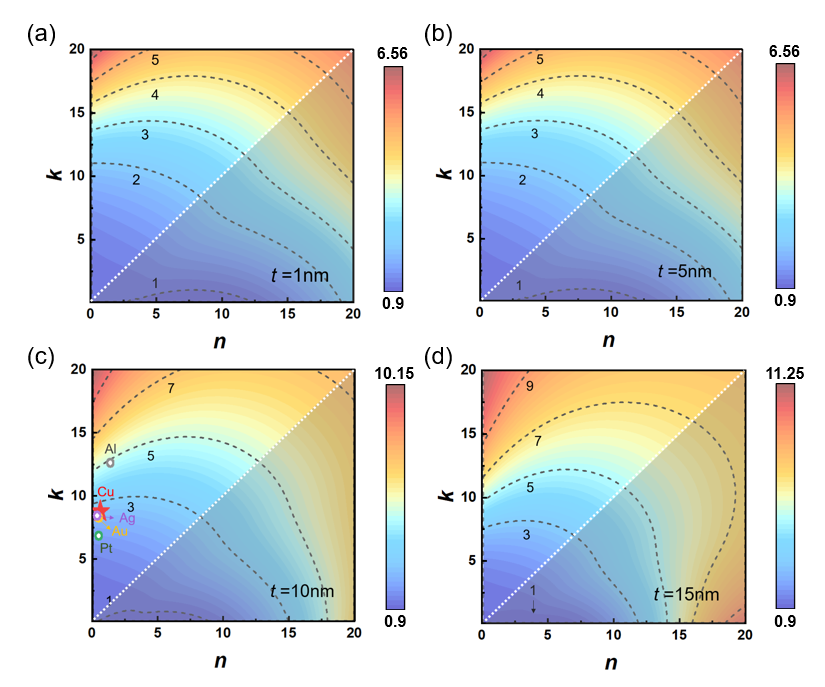


**Figure S3**. Universality of absorption enhancement in the QARC structure across different optical constants and metal thicknesses. Absorption enhancement calculated as a function of the complex refractive index *n*+*iκ* of the metal layer for different metal thicknesses: (a) *t* = 1 nm, (b) *t* = 5 nm, (c) *t* = 10 nm, and (d) *t* = 15 nm at the wavelength of 1300 nm.


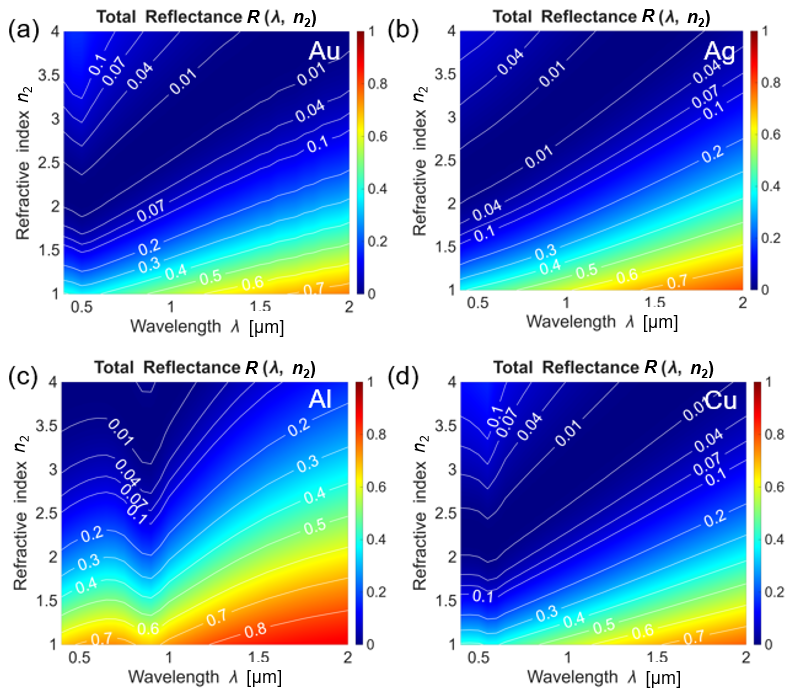


**Figure S4**. Calculated reflectance maps of QARC structures as functions of the overlayer refractive index *n*_2_​ and wavelength λ, assuming a metal thickness of 10 nm for (a) gold, (b) silver, (c) aluminum, and (d) copper.


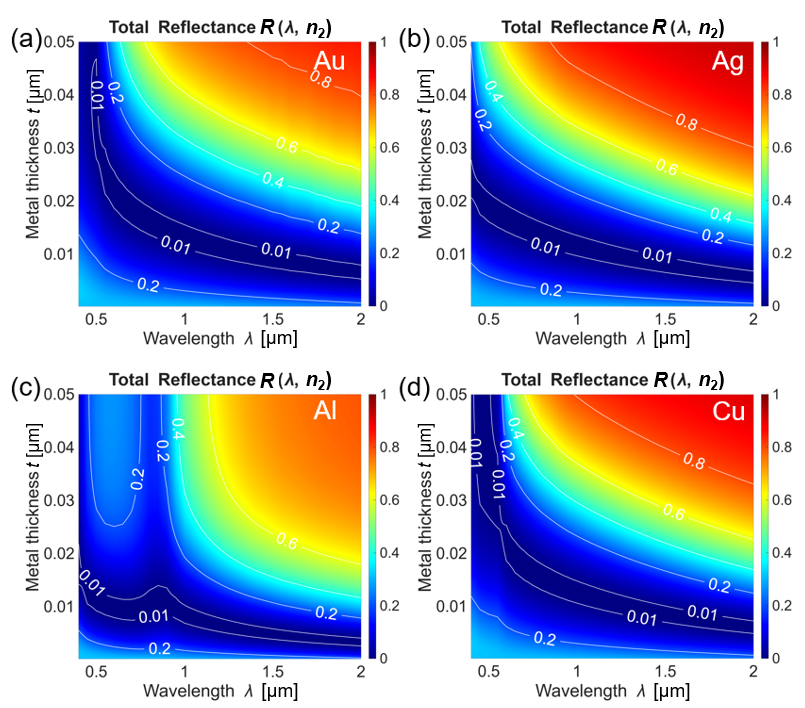


**Figure S5**. Calculated reflectance maps of QARC structures as functions of the metal thickness *t*​ and wavelength λ, assuming an overlayer refractive index of 3.5 for (a) gold, (b) silver, (c) aluminum, and (d) copper.


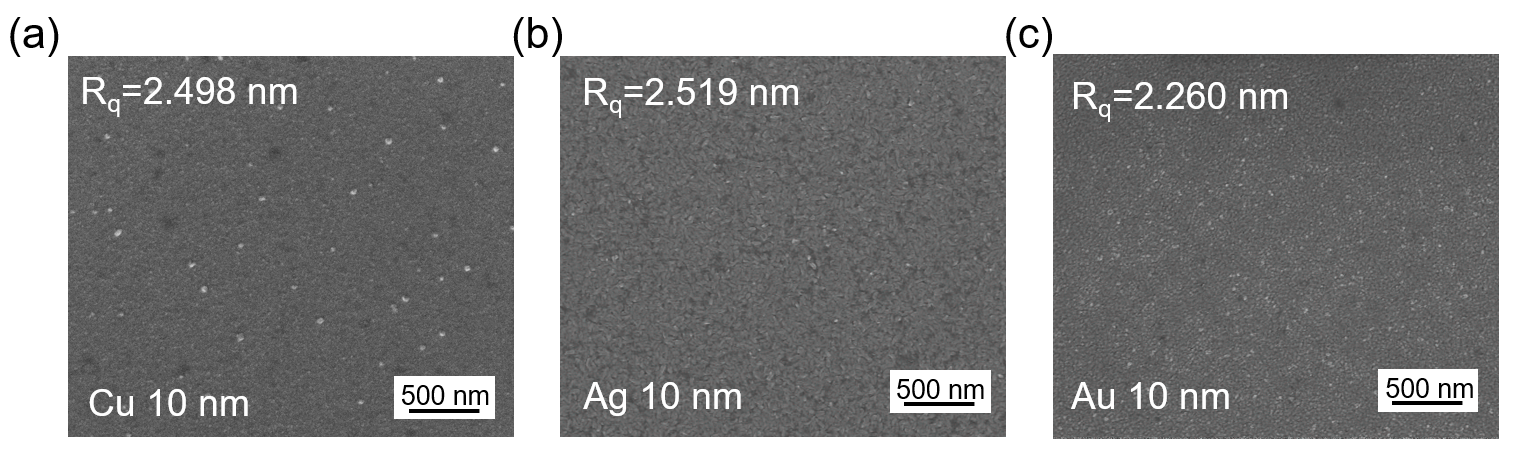


**Figure S6.** Surface morphology and roughness of 10-nm ultrathin metal films. (a) Cu. (b) Ag. (c) Au.


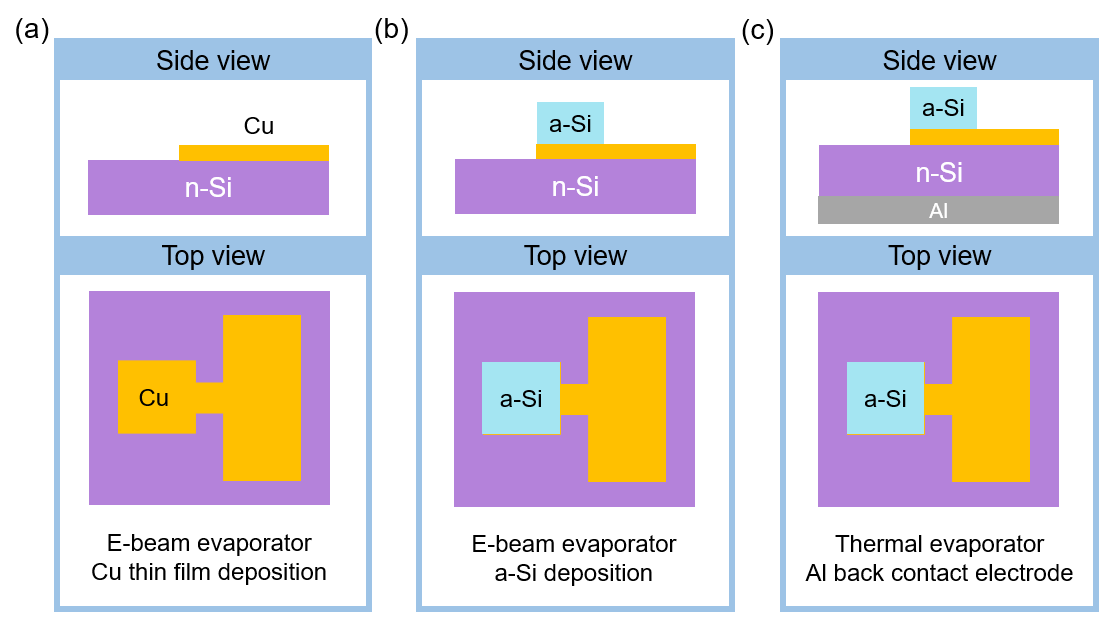


**Figure S7.** Fabrication process of the CMOS-compatible silicon hot-carrier photodetector. (a) Deposition of an ultrathin Cu electrode on n-type Si using an e-beam evaporator. (b) Deposition of the high-index a-Si layer on top of the Cu/Si structure via e-beam evaporation. (c) Formation of the Al back-contact electrode using a thermal evaporator, completing the Cu/a-Si/n-Si stack.

**
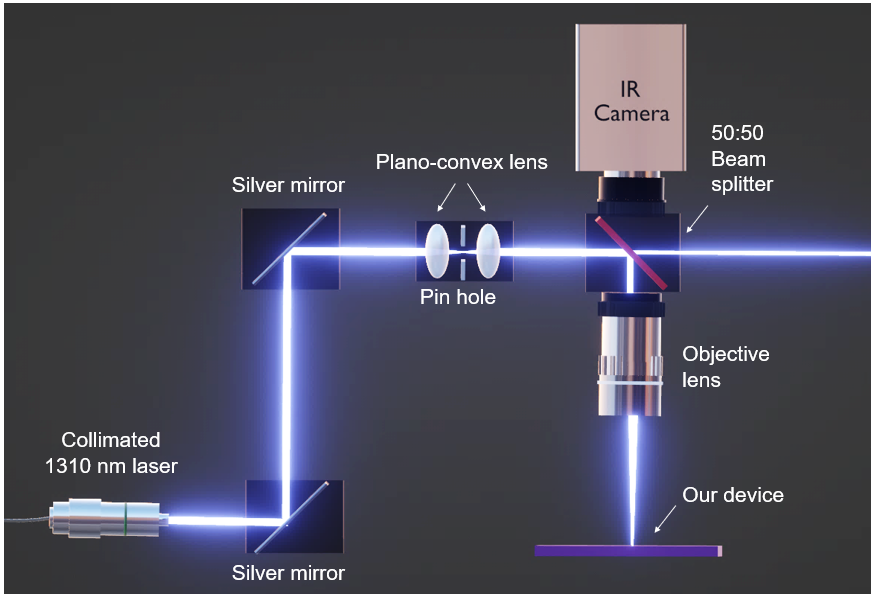
**

**Figure S8.** Optical measurement setup for evaluating the silicon hot-carrier photodetector. A collimated 1310 nm laser is guided through a series of silver mirrors and focused using a plano-convex lens pair with a pinhole for spatial beam filtering. The conditioned beam is then directed into a 50:50 beam splitter, where one path is monitored by an IR camera for real-time beam profiling, while the transmitted path is focused onto the device under test using an objective lens. This setup enables precise excitation, alignment, and SWIR photocurrent characterization of the fabricated hot-carrier photodetector.


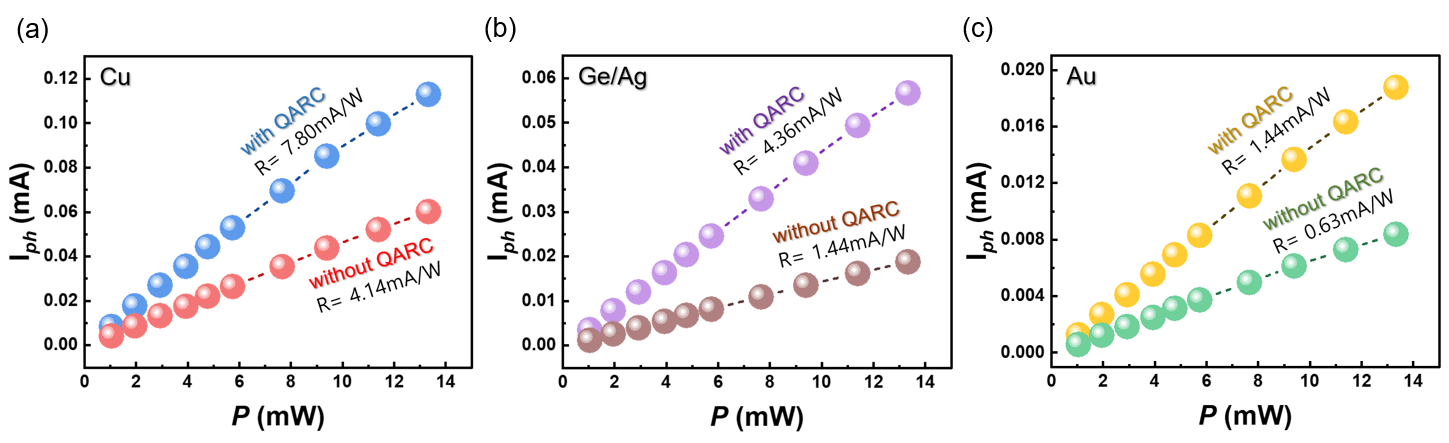


**Figure S9.** Photocurrent (*I*_ph_) as a function of incident optical power (*P*) for silicon hot-carrier photodetectors employing different metal electrodes. (a) Cu, (b) Ge/Ag, and (c) Au, with and without the QARC structure at zero bias. The solid lines represent linear fits used to extract the responsivity (*R* = *I*_ph_/*P*). In all cases, a linear relationship between photocurrent and optical power is observed, confirming reliable responsivity extraction.

**
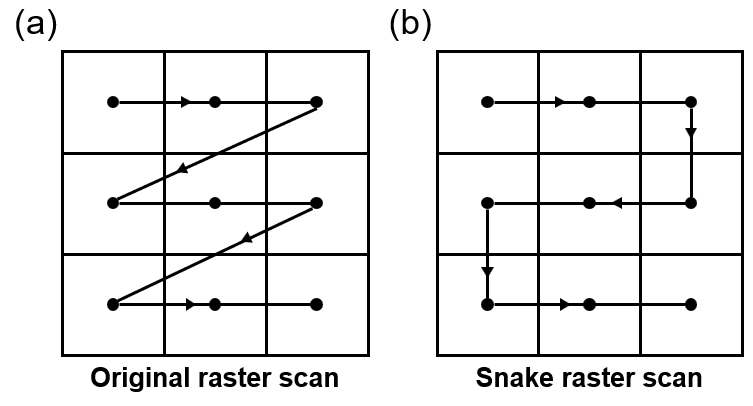
**

**Figure S10.** Raster scanning strategies. (a) Standard raster scan with unidirectional line acquisition and full flyback at each row. (b) Snake raster scan with alternating scan directions, reducing flyback time and improving acquisition efficiency.

**
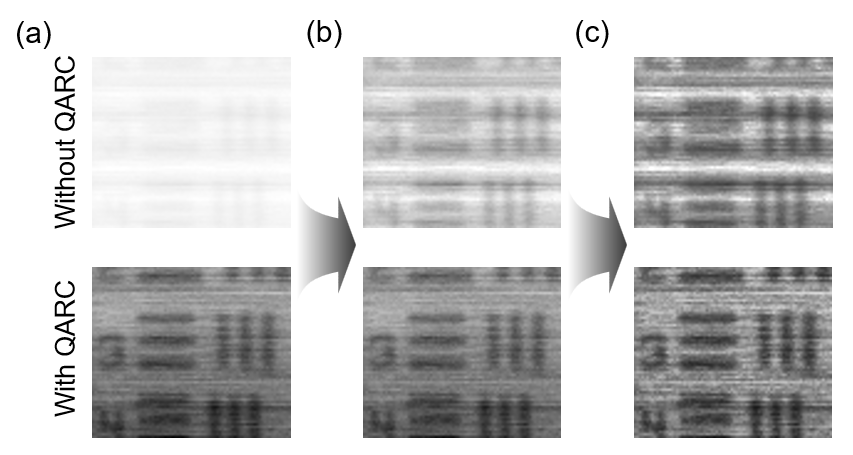
**

**Figure S11.** Stepwise processing of SWIR images from devices without and with QARC. (a) Images after global percentile-based normalization on a shared intensity scale. (b) Images after weak row-wise banding suppression. (c) Final images after soft CLAHE enhancement, highlighting the improved structural contrast of the QARC device.
